# Fecal Hyodeoxycholic Acid Is Correlated With Tylosin-Induced Microbiome Changes in Growing Pigs

**DOI:** 10.3389/fvets.2018.00196

**Published:** 2018-08-28

**Authors:** Michaela P. Trudeau, Yuyin Zhou, Fernando L. Leite, Andres Gomez, Pedro E. Urriola, Gerald C. Shurson, Chi Chen, Richard E. Isaacson

**Affiliations:** ^1^Department of Animal Science, University of Minnesota, St. Paul, MN, United States; ^2^Department of Food Science and Nutrition, University of Minnesota, St. Paul, MN, United States; ^3^Department of Veterinary and Biomedical Sciences, University of Minnesota, St. Paul, MN, United States; ^4^Department of Veterinary Population Medicine, University of Minnesota, St. Paul, MN, United States

**Keywords:** antibiotics, bile acids, metabolomics, microbiome, pigs

## Abstract

The changes in the gut microbiome play an important role in the promoting effects of antibiotics, such as tylosin, to the health, and productivity of farm animals. Microbial metabolites are expected to be key mediators between antibiotics-induced microbiome changes and growth-promoting effects. The objective of this study was to extend the identification of tylosin-responsive microbes to the identification of tylosin-responsive metabolites in growing pigs. The feeding trial was conducted on a commercial farm using two pens of pigs fed diets with and without tylosin (40 mg/kg of diet). Fecal samples were collected from 10 pigs per pen at weeks 10, 13, 16, 19, and 22 of age, and subsequently analyzed using liquid chromatography-mass spectrometry (LC-MS) analysis. The multivariate model of LC-MS data showed that time-dependent changes occurred in the fecal metabolome of both control and tylosin-treated pigs. More importantly, the metabolomic profiles were similar between the tylosin treatment and control groups in weeks 10 and 22, but diverged during weeks 13–19. Subsequent analyses of the fecal metabolites contributing to the separation of two groups of pigs showed that hyodeoxycholic acid (HDCA), together with tylosin and its metabolites in feces, was greatly increased during weeks 13–19 (*P* < 0.05) in the group of pigs fed tylosin. The integration of current metabolomics data and the microbiome data from a previous study revealed the consistency between HDCA and a specific genus of microbes in the *Clostridia* family. Further studies are required to determine the causative relations between tylosin-elicited changes in HDCA and the microbiome as well as the role of HDCA in the growth promoting effects of tylosin.

## Introduction

Current food animal production systems have been able to supply animal products (e.g., milk, eggs, meat) at lower cost than ever before. Likewise, efficiency of food animal production is greater in modern production systems than in the past, while also decreasing environmental impact (1). Modern farms attained such efficiencies in productivity in part because of the implementation of technologies such as utilization of antibiotics as growth promoters. Sub-therapeutic levels of antibiotics in feeds have been used in swine and poultry diets since the 1940s to improve growth performance of animals while also reducing sub-clinical disease (2, 3). However, the use of antibiotics also increases selective pressures responsible for the evolution of antibiotic resistant bacteria (4, 5). The One Health framework suggests that animal health is closely linked to human health and consequently, the use of antibiotic growth promoters increases the risk of antibiotic resistant bacteria in humans (6). Therefore, it is necessary to develop strategies that maintain and improve animal productivity while reducing the usage of antibiotics in the production of livestock.

The mechanism(s) whereby antibiotics improve growth and efficiency of pigs is still not completely understood, making it difficult for nutritionists, veterinarians, and food animal producers to identify antibiotic alternatives that can produce similar improvements in growth performance without using sub-therapeutic levels of antibiotics. Early experiments in poultry showed that germ-free chicks fed sub-therapeutic levels of antibiotics did not have improved growth compared to the ones fed control diets, indicating that the microbiome plays a significant role in the growth promotion process (7). The microbiome affects numerous physiological processes of animals including protection against some pathogens, development of the immune system and stimulation of immune responses, development of the epithelium, nutrient digestion, and nutrient metabolism (8). Because of the multiple roles ascribed to the microbiome in animals and the complexity of the composition of the microbiome, it has been difficult to define specific mechanisms of antibiotic growth promotion. To fully understand the impact of sub-therapeutic levels of antibiotics on animal growth, research is needed that integrate growth with the metabolome.

Previous studies identified that pigs fed the antibiotic tylosin, had prominent shifts in their fecal microbiome in both abundant and less abundant species compared with the pigs fed an antibiotic-free control diet (9). These results also showed that the composition of the microbiome converged over time, and tylosin appeared to increase the rate at which the microbiome matured. We hypothesized that this shift in microbiome maturation and ultimate convergence would also be represented in the functionality of the microbiome, especially the production of specific bacterial metabolites (10). The objective of this study was to determine tylosin-induced changes in the fecal metabolome of growing pigs and also to correlate these metabolic changes with tylosin-induced changes in the microbiome for a better understanding of the mechanisms mediating antibiotic growth promotion.

## Materials and methods

### Animal experiment

The animals were housed in conventional confinement facilities on a commercial farm located in southwestern Minnesota for the duration of the experiment [farm 2 in (9)]. Only samples collected from farm two of the experiment previously reported by Kim et al. were used for further analysis (2012). Two pens containing 50 pigs each were used in the experiment. Ten pigs in each pen were randomly chosen, ear tagged for identification and were sampled throughout the sampling period. Pigs in one pen received tylosin in their feed at a concentration of 40 mg/kg beginning at 10 weeks of age and continuing for 12 weeks. Tylosin was chosen as the antibiotic because of its frequent use for growth promotion in the swine industry. The second pen of pigs served as a control and pigs were fed the same feed except that tylosin was not included in it. None of the pigs were given any additional antimicrobials through the duration of the experiment, and all pigs were fed the same standard commercial corn-soybean meal diet. Fresh feces were collected directly from the rectum of the 20 pigs at 10, 13, 16, 19, and 22 weeks of age. Samples were stored at −80°C until analysis. The stability of bile acids in fecal samples after long-term storage has been demonstrated in a previous study (11). This study was approved by the Institutional Animal Care and Use Committee (IACUC) of the University of Minnesota (Protocol 0705A09361).

### Metabolomics analysis

#### Chemicals and reagents

LC–MS-grade water and acetonitrile (ACN) were purchased from Fisher Scientific (Houston, TX); triphenylphosphine (TPP) and 2- hydrazinoquinoline (HQ) from Alfa Aesar (Ward Hill, MA); 2,2′-dipyridyl disulfide (DPDS) from MP Biomedicals (Santa Ana, CA); tylosin tartrate from Ark Pharm (Arlington Heights, IL); acetic acid-d_4_ from Sigma-Aldrich (St. Louis, MO); glycocholic acid-^13^C_1_ from C/D/N Isotopes (Quebec, Canada). The metabolite standards used for structural confirmation were from Sigma-Aldrich, Fisher Scientific, AlfaAesar, Ark Pharm (Libertyville, IL), respectively.

#### Fecal sample preparation

Fifty mg of pig fecal samples were mixed with 50% aqueous ACN containing 5 μM glycocholic acid-^13^C_1_ in 1:10 (w/v) ratio and sonicated for 10 min. The samples were then subjected to further mixing using a vortex mixer and then were centrifuged at 18,000 × g at 4°C for 10 min to obtain fecal sample extracts. The extracts were stored at −80°C prior to further analysis.

#### Derivatization of short-chain fatty acids (SCFAs) in fecal samples

Short-chain fatty acids (SCFAs) in the pig fecal samples were derivatized with HQ prior to LC-MS analysis using a modification of (12). Two microliters of the extract supernatant were mixed with 70 μL of acetonitrile containing 7.5 μM acetic acid-d_4_, 10 μL DPDS, 10 μL TPP, and 10 μL HQ. The mixture was incubated at 60°C for 30 min, chilled on ice, and mixed with 100 μL H_2_O. The mixture was then centrifuged at 18,000 × *g* for 10 min. Five microliters of the supernatant were injected into the UPLC system.

#### LC–MS analysis

Fecal extracts were analyzed in both non-derivatized form and derivatized form. Non-derivitized fecal extracted were separated a BEH C18 column (Waters, Milford, MA) using a mobile phase gradient containing 0.1% formic acid (A) and ACN containing 0.1% formic acid (B). For SCFAs analysis, HQ-derivitized fecal samples were separated a BEH C18 column (Waters, Milford, MA) using a mobile phase gradient containing 2 mM ammonium acetate and 0.05% acetic acid, v/v (A), and H_2_O/ ACN = 5:95, v/v, containing 2 mM ammonium acetate and 0.05% acetic acid, v/v (B). The LC eluant was introduced into a Xevo-G2-S quadrupole time-of-flight mass spectrometer (Waters) for accurate mass measurement and ion counting. Capillary voltage and cone voltage for electrospray ionization was maintained at 0.1 kV and 5 V for negative-mode detection, and at 3 kV and 30 V for positive-mode detection. Source temperature and desolvation temperature were set at 120 and 350°C, respectively. Nitrogen was used as both cone gas (50 L/h) and desolvation gas (800 L/h), and argon was used as collision gas. For accurate mass measurement, the mass spectrometer was calibrated with sodium formate solution with a mass-to-charge ratio (m/z) of 50–1,000 and monitored by the intermittent injection of the lock mass leucine enkephalin ([M + H]^+^ = 556.2771 *m/z* and [M - H]^−^ = 554.2615 *m/z*) in real time. Mass chromatograms and mass spectral data were acquired and processed by MassLynx software (Waters) in centroided format. The concentration of individual compounds was determined by calculating the ratio between the peak area of compound and the peak area of internal standard and fitting with a standard curve using QuanLynx software (Waters).

#### Chemometric analysis and biomarker identification

The chromatographic and spectral data of fecal extracts were deconvoluted by MarkerLynx software (Waters). A multivariate data matrix containing information on sample identity, ion identity (retention time and *m/z*), and ion abundance was generated through centroiding, deisotoping, filtering, peak recognition, and integration. The intensity of each ion was calculated by normalizing the single-ion counts (SIC) vs. the total-ion counts (TIC) in the whole chromatogram. The data matrix was further exported into SIMCA-P+ software (Umetrics, Kinnelon, NJ) and transformed by *Pareto* scaling, and then analyzed by unsupervised principal component analysis (PCA), supervised partial least squares-discriminant analysis (PLS-DA), and supervised orthogonal partial least squares-discriminant analysis (OPLS-DA). Major latent variables in the data matrix were described in a scores scatter plot of the established multivariate model. Metabolites affected by tylosin were identified by analysis of ions contributing to the separation of tylosin and control samples in the loadings plot the models. The chemical identities of compounds of interest were determined by accurate mass measurement, elemental composition analysis, MSMS fragmentation, and comparisons with authentic standards if available.

### Microbiome correlation analysis

Isolation of DNA, PCR amplicon production, sequencing, and analysis were all completed and analyzed previously (9). Since the microbiome data analysis only used pooled data, the average relative abundance of metabolites for each time point and treatment were used in this calculation. This allowed the data sets to be equally compared for the correlations analysis. The average values for the metabolomics samples were only used for the correlation analysis. Weighted Bray-Curtis beta diversity metrics were calculated using the vegan package in the statistical software R (13). The dissimilarity distance matrix for both the microbiome and metabolome data was calculated after relative abundance transformations to account for non-normal distributions. Correlations between microbiome and metabolome data were calculated using the mantel test and procrustes analyses, also within the vegan R package (13). A multiple correlation analysis approach, based on Spearman correlation coefficients and adjusted using false discovery rate (fdr) methods for multiple testing using the microbiome R package (14), was also conducted to assess how the abundance of identified bacterial taxonomic units covaried with the abundance of identified bile acid metabolites.

## Results

### Metabolomic comparisons

**Figure 1:**
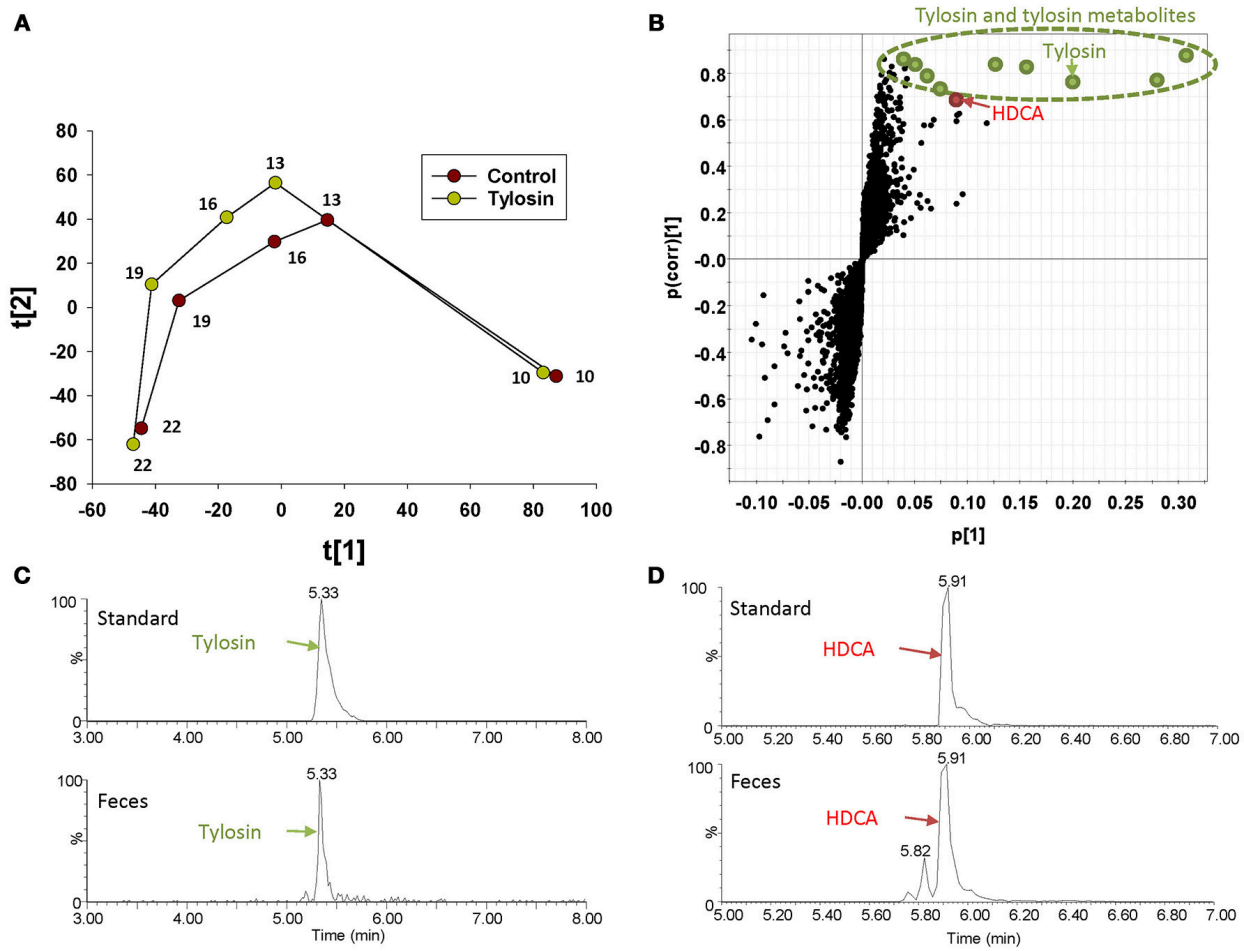
Identification of fecal metabolites induced by tylosin treatment through LC-MS-based metabolomics. **(A)** Scores plot of a PLS-DA model on fecal samples from the tylosin-treated and control pigs. The t(1) and t(2) values represent the scores of each data point in the principal component 1 and 2 of the model, respectively. These values are the averages of 10 pigs under the same treatment at weeks 10, 13, 16, 19, and 22. **(B)** S-plot of an OPLS model on week 13–19 control and tylosine treatment samples. The fecal metabolites contributing to the separation of two groups of pigs are labeled. The p(1) axis represents the magnitude of the fecal ions. The p(corr)(1) axis represents the correlation of the ions toward the predictive variation induced by tylosin treatment. **(C)** Extracted chromatograms of tylosin standard and a fecal sample. **(D)** Extracted chromatograms of HDCA standard and a fecal sample.

The distribution of fecal samples in the score plot of a PLS-DA model showed that time-dependent changes in the fecal metabolome occurred in both control and tylosin-treated pigs (Figure 1A and Figure S1). Between the two treatment groups, the metabolome profiles were comparable at 10 and 22 weeks of age, but different during weeks 13–19 (Figure 1A and Figure S1). The metabolites contributing to the separation between control and tylosin groups in 13, 16, and 19 weeks of pigs were defined in the S-plot of a OPLS-DA model (Figure 1B). As expected, tylosin and its metabolites contributed to the separation of two groups of pigs in the models (Figure 1C). More importantly, HDCA, a bile acid, was identified as another prominent marker associated with tylosin feeding (Figure 1D).

**Figure 2:**
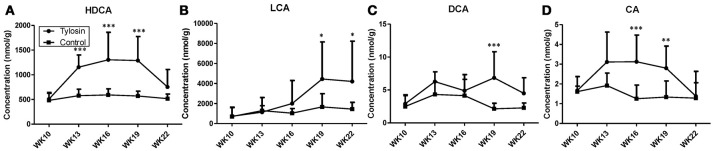
Concentrations of bile acids in fecal samples from control and tylosin-treated pigs from week 10 to week 22. **(A)** HDCA. **(B)** LCA. **(C)** deoxycholic acid (DCA). **(D)** CA. Values are mean ± S.D. (*P ≤ 0.05; **P ≤ 0.01; ***P ≤ 0.001).

**Figure 3:**
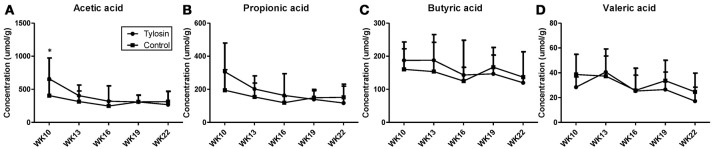
Concentrations of SCFAs in fecal samples from control and tylosin-treated pigs from week 10 to week 22. **(A)** Acetic acid. **(B)** Propionic acid. **(C)** Butyric acid. **(D)** Valeric acid. Values are mean ± S.D. (*P ≤ 0.05).

Following the observation of HDCA as a tylosin-responsive metabolite, the levels of bile acids in feces were quantified. Based on their concentrations, HDCA and lithocholic acid (LCA) are major bile acids while deoxycholic acid (DCA) and cholic acid (CA) are minor ones in pig feces (Figures 2A–D). More importantly, the results showed that the concentrations of fecal bile acids were relatively stable in the control group between week 10 and 22, but significantly and differently affected by tylosin in the treatment group (Figures 2A–D). HDCA and CA shared a comparable time-course profile, since the concentrations of both bile acids were elevated by tylosin during weeks 13 and 19, but became comparable to the controls on week 22 (Figures 2A,D). In contrast, DCA, and LCA were only increased by tylosin treatment during weeks 19 and 22 (Figures 2B, C). Besides bile acids, short chain fatty acids (SCFA) in these fecal samples, including acetic acid, propionic acid, butyric acid, and valeric acid, were also quantified. The acetic acid concentrations were different (*P* < 0.05) between the control and treatment group at the 10 weeks of age (Figure 3A). This pre-existing difference between groups cannot be simply explained by tylosin treatment because the antibiotic was only added a few hours before the fecal samples were collected. Aside from this difference at a single time point, there were no differences in the concentration of any SCFA between the treatment and control group (Figures 3A–D).

### Comparison of metabolome and microbiome

**Figure 4:**
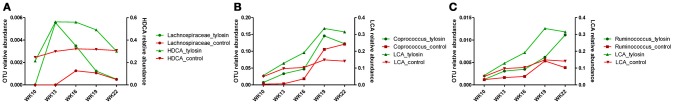
Comparison of relative abundance for significantly correlated bacterial species over time for HDCA **(A)** and LCA **(B,C)**.

There was a positive correlation (*r* = 0.78, *P* = 0.001) between the microbiome composition and metabolomic patterns at all-time points for both treatments, revealed by a Mantel test. Procrustes analysis based on the Spearman method further confirmed this correlation (correlation in a symmetric Procrustes rotation = 0.88, *P* = 0.001, m12 squared = 0.22). Multiple correlation analysis was also used to detect associations between identified metabolites and OTUs in the microbiome. Significant correlations (*Q* < 0.05) were observed between bile acid metabolites and limited OTUs (Table S1). HDCA is the most common bile acid in pigs, and was associated with the abundance of the family *Lachnospiraceae*, which belong to order of Clostridiales. LCA was associated with *Coprococcus* and *Ruminococcus* in both treatment and control groups (Table 1). To further evaluate these relationships over time, the concentrations of bile acid metabolites were plotted against the abundances of their correlated bacterial species (Figure 3). For LCA, even though metabolite concentration between treatment and control deviated in later weeks, the abundance of *Coprococcus* remained similar between groups but increased over time. The association between LCA and *Ruminococcus* appeared to be more direct, meaning the concentration of LCA increased over time in the tylosin treatment group, as did the abundance of *Ruminococcus* (Figure 4). There was an initial increase in the levels of *Lachnospiraceae* and HDCA in pigs fed tylosin, but at subsequent time points, the levels of both decreased to match the control group at 22 weeks (Figure 4).

**Table 1:** Significant correlations (Q < 0.05) between tylosin-responsive bile acids and tylosin-responsive families in swine feces.

**Taxa**	**Metabolite**	**r-value**	**Q-value^a^**
k__Bacteria.p__Firmicutes.c__Clostridia.o__Clostridiales.f__Lachnospiraceae.g__*Coprococcus*.s__.60	LCA	0.939394	<0.0001
k__Bacteria.p__Firmicutes.c__Clostridia.o__Clostridiales.f__Ruminococcaceae.g__*Ruminococcus*.s__.102	LCA	0.951515	<0.0001
k__Bacteria.p__Firmicutes.c__Clostridia.o__Clostridiales.f__Lachnospiraceae.79	HDCA	0.963263	0.0313

a *Adjusted for multiple comparisons in the model through false discovery rate*.

## Discussion

Because metabolites can function as energy carriers and signaling initiators of the growth and wellbeing of host and gut microbes, metabolomic analysis could provide useful insights on the connections between growth promoting effects and microbiome modulating effects of antibiotics. In this study, the composition of the fecal metabolome was similar between tylosin-treated pigs and control pigs at weeks 10 and 22, but different at weeks of 13, 16, and 19 of age (Figure 1). This observation resembles our previous observation on the fecal microbiome of these pigs, because the differences in the distribution and quantity of microbes between the control and tylosin treated group were also observed at weeks 13, 16, and 19, but not weeks 10 and 22 (9). This phenomenon suggested that tylosin might cause the microbiome to mature at a faster rate and then stabilize by week 22 (9). Interestingly, this “maturation” of the microbiome was observed in a different set of animals and samples (farm 1) compared to the samples used for the metabolomics analysis presented in this paper (farm 2) (9). The authors explained this variation in microbiome between farms as a technical issue from more in-depth sequencing on farm two, variation in the microbiome between farms that could respond differently to antibiotics, or inaccuracy of the maturation hypothesis. Though this pattern was not as clear in the microbiome on farm two, our results from metabolomic analysis still support this hypothesis, showing similar metabolite compositional patterns between groups at 10 and 22 weeks, with convergence of the metabolome profiles at 22 weeks. When evaluating this phenomenon from an ecological perspective, it has been proposed that the microbiome is always driven to return to a stable state, even after the impact of a stressor, such as antibiotic exposure (15, 16). Our results suggest that the functionality of the microbiome may also follow this pattern, as reflected by convergence of metabolome profiles between the tylosin treated pigs and control pigs.

Because of the role that the microbiome plays in converting primary bile acids to secondary bile acids, we hypothesized that the concentration of secondary bile acids would be altered after exposure to tylosin (17). Previous studies have shown that antibiotics can impact secondary bile acid secretion in humans and rats (18, 19). Furthermore, previous research has also identified that variation in the gut microbiome between germ-free and conventional mice impacts primary bile acid synthesis in the liver through interactions between gut microbes and the nuclear receptor Farnesoid X receptor (20). For this reason, it was also hypothesized that we would observe variations in primary bile acid secretion between the tylosin treatment and the control group. Although the concentration of CA (primary bile acid) was different between treatment and control group, this difference was only present for weeks 16 and 19. It is still unknown which species of bacteria are most involved in the regulation of the Farnesoid X receptor pathway, and we were unable to confirm if antibiotic induced changes in the microbiome impacted this pathway.

Our data also showed differences in HDCA concentration between the tylosin treatment and control group. Because HDCA is produced in germ-free pigs, it has been considered to also be a primary bile acid (21). However, previous research has also demonstrated that a healthy microbiome is capable of producing significant amounts of HDCA, indicating it could also be considered to be a secondary bile acid (22). Based on these conflicting results, it is unclear if HDCA should be considered a primary or secondary bile acid. For this reason, it is difficult to determine which mechanism may be impacting the increased concentration of HDCA in our experiment (i.e., action from the microbiome or interaction with the liver and primary bile acid production). We also found differences in the concentration of LCAs between treatments, which is another secondary bile acids in pigs. The abundance of bacterial class *Clostridia* have been shown to be correlated with intestinal secondary bile acids (23). It has also been reported that bacteria from the family *Clostridia* plays a critical role in bile acid deconjugation (24). In our experiment, we identified a significant, positive correlation with the secondary bile acids LCA and HDCA that were associated with three bacterial species in the class *Clostridia* (Class *Coprococcus, Ruminococcus*, and *Lachnospiraceae*). Thus, we suggest that feeding sub-therapeutic levels of Tylosin may lead to increases in the abundance of *Clostridia*, and ultimately increasing the production of secondary bile acids. It is also worth noting that the majority of previous studies that have reported changes in bile acid metabolism, fed greater antibiotic doses compared to sub-therapeutic levels of tylosin fed in the current study (18, 23, 25). Our experiment found similar alterations in the microbiome, leading to consequential changes in the animals' metabolome, even with a relatively low dose of tylosin.

SCFA are a major group of microbial metabolites in the large intestine (26). Influences of antibiotic exposure on SCFA production have been observed in both human and animal studies. For example, the concentrations of SCFA in feces were reduced by the 6-day treatment of a variety of antibiotics in healthy human subjects (27), while feeding sub-therapeutic levels of antibiotics increased concentrations of SCFA in cecum of treated mice (28). In contrast to these observations, no difference in fecal SCFA concentrations was observed between control and tylosin-treated pigs in this study. It is possible that bacteria responsible for SCFA production might not be sensitive to the dose of tylosin in this study. Tylosin is macrolide-class broad spectrum antibiotic commonly used for its activity against gram-negative bacteria, but is also effective against a select number of gram-positive bacteria (29) Specific gram-positive bacteria from the families *Propionibacteriaceae, Bifidobacteriaceae*, and *Veillonellaceae* have been shown to play a role in SCFA production (30). It is possible that tylosin did not have a bacteriostatic effect on some of these bacteria, allowing them to continue SCFA production without major changes.

One of the main limitations of our experiment was our inability to correlate the change in bile acid synthesis to a change in growth performance or health of pigs, because body weights, mortality, and morbidity data were not collected during the experiment. There is currently limited research available that has reported a direct change in growth performance of swine as a result of increased bile acid synthesis, but some previous research suggests that the mechanism for growth promotion when feeding antibiotics are due to changes in bile biotransformation (31, 32). However, this proposed mechanism suggests that increased bile acid secretion decreases average daily gain in the animal, which has been demonstrated in swine with LCA (25). Our results showed that the concentrations of LCA and other bile acids increased in pigs fed tylosin, which suggests that these differences may be specific to tylosin. Various antibiotics target different types of bacteria, which suggests that the mechanisms of growth promotion through modulation of the gut microbiome will vary between antibiotics used (3, 20, 23). Without growth performance data being available from this experiment, we are unable to determine the impact of the altered bile acid concentrations on growth of these pigs.

In conclusion, inclusion of sub-therapeutic levels of tylosin in the diet of growing pigs impacted bile acid concentration in the feces, but this change tended to diminish in subsequent time periods. These observations warrant further investigation to better understand the role of bile acids in growth and development of pigs, and whether these observations may be correlated with the mechanisms of growth promotion when supplementing diets with sub-therapeutic levels of antibiotics for pigs.

## Author contributions

MT, YZ, FL, CC, and RI all contributed equally to this work. MT, FL, RI, GS, and PU conceived and designed the experiments. YZ and CC performed the experiments. MT, YZ, and AG analyzed the data. CC contributed reagents, materials, analysis tools. MT, YZ, FL, AG, CC, PU, GS, CC, and RI wrote the paper.

### Conflict of interest statement

The authors declare that the research was conducted in the absence of any commercial or financial relationships that could be construed as a potential conflict of interest.
