# Supplementary figures and images for: Fecal Hyodeoxycholic Acid Is Correlated With Tylosin-Induced Microbiome Changes in Growing Pigs
Source: Front Vet Sci. 2018 Aug 28;5:196. doi: 10.3389/fvets.2018.00196 (PMC6121748; doi:10.3389/fvets.2018.00196)

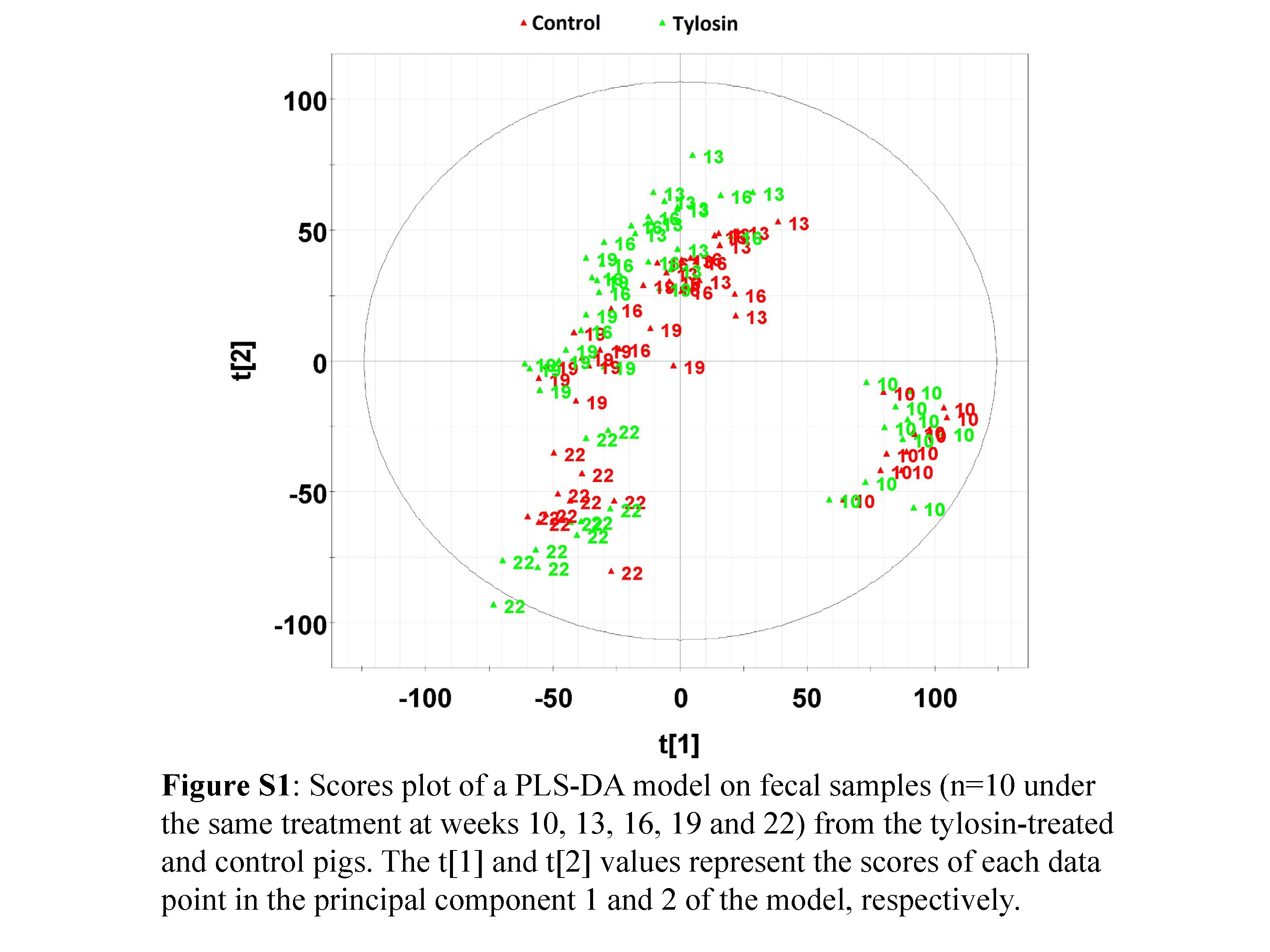

Supplement: Supplementary file 2 [file Image_1.jpg]
